# Metal-organic Framework-driven Porous Cobalt Disulfide Nanoparticles Fabricated by Gaseous Sulfurization as Bifunctional Electrocatalysts for Overall Water Splitting

**DOI:** 10.1038/s41598-019-56084-9

**Published:** 2019-12-20

**Authors:** In-Kyoung Ahn, Wonhyo Joo, Ji-Hoon Lee, Hyoung Gyun Kim, So-Yeon Lee, Youngran Jung, Ji-Yong Kim, Gi-Baek Lee, Miyoung Kim, Young-Chang Joo

**Affiliations:** 1 Department of Materials Science & Engineering, Seoul National University, Seoul, 08826 Republic of Korea; 2 Materials Center for Energy Convergence, Surface Technology Division, Korea Institute of Materials Science (KIMS), Changwon, Gyeongnam 51508 Republic of Korea; 3 Research Institute of Advanced Materials (RIAM), Seoul National University, Seoul, 08826 Republic of Korea

**Keywords:** Electrocatalysis, Electrochemistry

## Abstract

Both high activity and mass production potential are important for bifunctional electrocatalysts for overall water splitting. Catalytic activity enhancement was demonstrated through the formation of CoS_2_ nanoparticles with mono-phase and extremely porous structures. To fabricate porous structures at the nanometer scale, Co-based metal-organic frameworks (MOFs), namely a cobalt Prussian blue analogue (Co-PBA, Co_3_[Co(CN)_6_]_2_), was used as a porous template for the CoS_2_. Then, controlled sulfurization annealing converted the Co-PBA to mono-phase CoS_2_ nanoparticles with ~ 4 nm pores, resulting in a large surface area of 915.6 m^2^ g^−1^. The electrocatalysts had high activity for overall water splitting, and the overpotentials of the oxygen evolution reaction and hydrogen evolution reaction under the operating conditions were 298 mV and −196 mV, respectively, at 10 mA cm^−2^.

## Introduction

To replace depleting fossil fuels, the development of environmentally-friendly and sustainable energy sources has been strongly urged. Among alternative energy sources, hydrogen-based energy sources are one of the most promising candidates due to their outstanding energy density, high energy conversion efficiency, and environmental friendliness^1–4^. Although there are a variety of technologies to produce hydrogen energy, electrochemical water splitting is one of the most promising choices due to commercial aspects^5–10^.

Electrochemical water splitting consists of two half reactions, namely the oxygen evolution reaction (OER) and the hydrogen evolution reaction (HER). Although noble metal electrocatalysts, such as Pt (for HER) and Ru- or Ir-based materials (for OER), show excellent catalytic performance, they are difficult to apply on a large scale because of cost issues. Thus, active electrocatalysts based on earth-abundant elements, such as transition metal compounds, have been extensively studied to replace noble metals^11,12^. Transition metal oxides^13,14^, hydroxides^15,16^, carbide^17^, nitride^18^, phosphide^19^, and sulfides^20–22^ have been studied as OER electrocatalysts under alkaline conditions, and transition metal sulfides^20–22^, phosphides^6,7^, carbonitride^23^, and selenides^24^ have been studied as HER electrocatalysts under acidic conditions. In regard to OER and HER performance, the effects of activation and optimization of electrocatalysts have recently been reported^17,18,23^. Furthermore, with a growing demand for simplicity and cost effectiveness, the need for bifunctional electrocatalysts, which operate in the same electrolyzer, is rapidly increasing^21,25,26^.

Cubic pyrite-phase transition metal dichalcogenides, such as FeS_2_^27,28^, NiS_2_^29^, and CoS_2_^26^, have been proposed as candidates for bifunctional electrocatalysts^30^. Among them, cobalt disulfide (CoS_2_) has been reported to exhibit high electrical conductivity^31^ and excellent activity for both the OER and the HER^9,26,32^. Since water splitting involves hydrogen and oxygen gas evolution reactions, porosity in the electrocatalysts is very important for improving their performance by facilitating gas emission and exposing active sites^26^. Therefore, to further increase the catalytic activity of CoS_2_, it is necessary to synthesize porous CoS_2_ with nanometer scale pores.

Metal-organic frameworks (MOFs), which comprise metal ions and organic linkers, can provide a large surface area with open structures, so they are used in many electrochemical applications, such as batteries, capacitors, and catalysts^33–35^. However, MOFs have a low electrical conductivity and are not suitable as electrocatalysts, which require a large current density for electrical efficiency.

Here, we fabricated mono-phase CoS_2_ with ~ 4 nanometer-scale pores as bifunctional water splitting electrical catalysts. Cobalt-based MOFs, namely a cobalt Prussian blue analogue (Co-PBAs, Co_3_[Co(CN)_6_]_2_), were used as a starting material. The Co-PBA was then sulfurized in *thermodynamically controlled conditions* to remove the organic linkers in the MOFs, resulting in pure and mono-phase CoS_2_. The synthetic process was predicted based on thermodynamics because cobalt sulfide has been reported in various phases^36^, where the phase depends on the S/Co ratio, such as CoS^37^, Co_9_S_8_^38^, and CoS_2_^39,40^. Thermodynamic calculations control the sulfur vapor pressure depending on the amount of cobalt-based starting materials before sulfurization. Such MOF-driven CoS_2_ nanoparticles still preserve the nonporous structure in the starting MOFs, so high electrocatalytic activities are achieved.

## Result and Discussion

### Designing of MOF-driven CoS_2_ electrocatalyst

**Figure 1:**
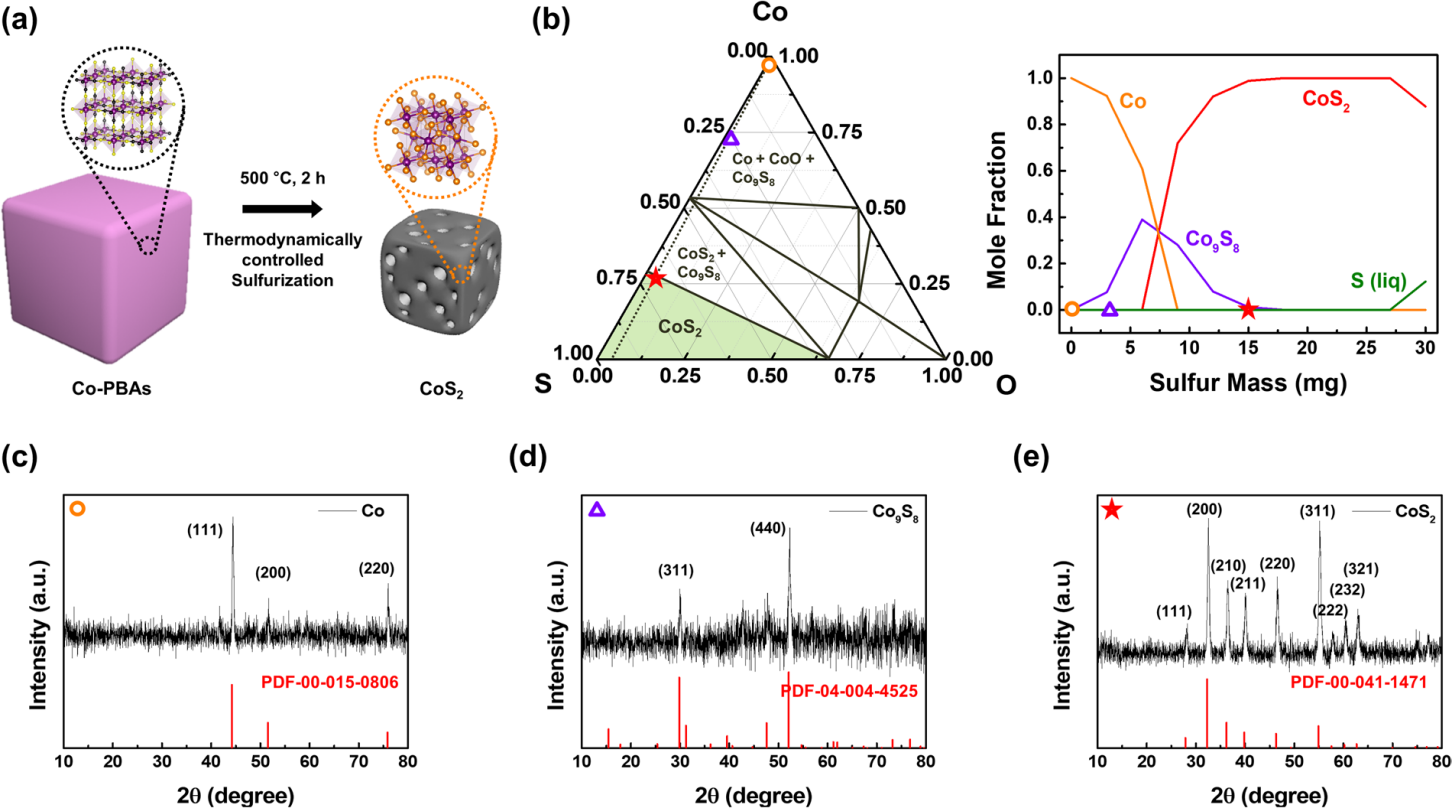
Summary of the fabrication of porous CoS_2_ and related thermodynamics calculations and its structural characterization. (**a**) Schematics of the synthesis of porous CoS_2_ through the sulfurization of Co-PBAs. (**b**) Ternary phase diagram at 500 °C for predicting the required composition to form the target phase and expected products depending on the amount of sulfur through thermodynamic prediction. (**c**–**e**) X-ray diffraction (XRD) analysis for verification of sulfurization under each condition. (**c**) Annealing for only Co-PBAs, (**d**) sulfurization of Co-PBAs with 3 mg sulfur (0.1 weight ratio of sulfur/Co-PBAs), and (**e**) sulfurization of Co-PBAs with 15 mg sulfur (0.5 weight ratio of sulfur/Co-PBAs).

Figure 1 shows the synthetic process before and after completion. In the first step, the amount of sulfur was controlled through the thermodynamic prediction, and then the sulfurization of the Co-PBAs was conducted under each condition using a thermal treatment. Before sulfurization, the morphology of the synthesized Co-PBAs was confirmed through field-emission scanning electron microscopy (FE-SEM) and transmission electron microscopy (TEM) images, as shown in Fig. S1. An angular shape is typically confirmed in PBAs, whose size distribution is usually from tens of nanometers to micrometers. Additionally, PBA particles were uniformly synthesized by adding sodium citrate. As proved in previous reports, dissolved citrate in the solution suppressed the nucleation of PBAs^41^.

For thermodynamically controlled sulfurization of Co-PBAs, components of the material and process parameters should be determined before the experiment. The synthesis of MOF-driven CoS_2_ is schematically shown in Fig. 1a. As a cobalt precursor, a Co-PBA was selected, and sulfur powder was used as a sulfur source. A closed system, comprising a sealed glass ampoule (Fig. S2), was used to ensure the loaded solid sulfur source was in a fully vaporized state for the thermodynamic calculations. Both were inserted into the glass ampoule, and the ampoule was fused to create a seal while the pressure inside was maintained at 0.1 Torr by rotary pump. Next, the ampoule was heated to 500 °C, which is higher than the boiling point of sulfur (445 °C). The sulfur inside was completely vaporized, and a reaction between the solid Co-PBA and gaseous sulfur took place. However, during such sulfurization processes, reaction products are dependent on the composition.

The Co-S-O ternary phase diagram at 500 °C was used to find the mole fraction of cobalt and oxygen for the sulfurization of the cobalt-based MOFs, and the determined value was set as the sulfur amount (left of Fig. 1b). Since C and N in Co-PBAs are vaporized to CN at 500 °C, the ternary phase diagram consists of Co-S-O components (Fig. S3). As the amount of sulfur content is increased, a cobalt sulfide phase with an increased S/Co ratio is synthesized. To set the exact sulfur amount, the ratio of each element (Co and O) was calculated. The moles of Co and O were fixed to the amount corresponding to 30 mg of Co-PBA in air 0.1 Torr in a 5 ml ampoule, illustrated as the dotted line in the phase diagram. Then, the amount of sulfur was the only parameter. Three experimental conditions for sulfur content were selected: 0 (0 mg), 0.1 (3 mg), and 0.5 (15 mg) weight ratios of sulfur/Co-PBAs, as marked in Fig. 1b as a circle, triangle, and star, respectively. The amount of reaction products at 500 °C according to the sulfur loading was calculated and is illustrated on the right in Fig. 1b. As the sulfur content increased, Co_9_S_8_ was produced at first, and then the amount of CoS_2_ increased. For a sulfur content with a ratio of more than 0.5 (15 mg), a CoS_2_ single phase can be obtained. However, when the sulfur content exceeded 0.9 (27 mg), the liquid sulfur phase remained.

Figure 1c–e shows the phase analysis of annealed Co-PBAs after the different processing conditions. XRD was conducted for phase analysis after each thermodynamically controlled condition was held for 2 hours. When a 0.5 ratio (15 mg) of sulfur was loaded with mono-phase CoS_2_, the target phase was synthesized, while a 0 ratio (0 mg) of sulfur synthesized the Co single phase and a 0.1 ratio (3 mg) of sulfur synthesized the Co_9_S_8_ phase. As a result of the XRD analysis, it was determined that annealing Co-PBAs without sulfur corresponds to the Co reference card in Fig. 1c, and sulfurization of the Co-PBAs in the condition represented by a triangle corresponds to the Co_9_S_8_ reference card in Fig. 1d. The XRD analysis in Fig. 1e clarified that CoS_2_ is the mono-phase.

### Structural characterization of MOF-driven CoS_2_

**Figure 2:**
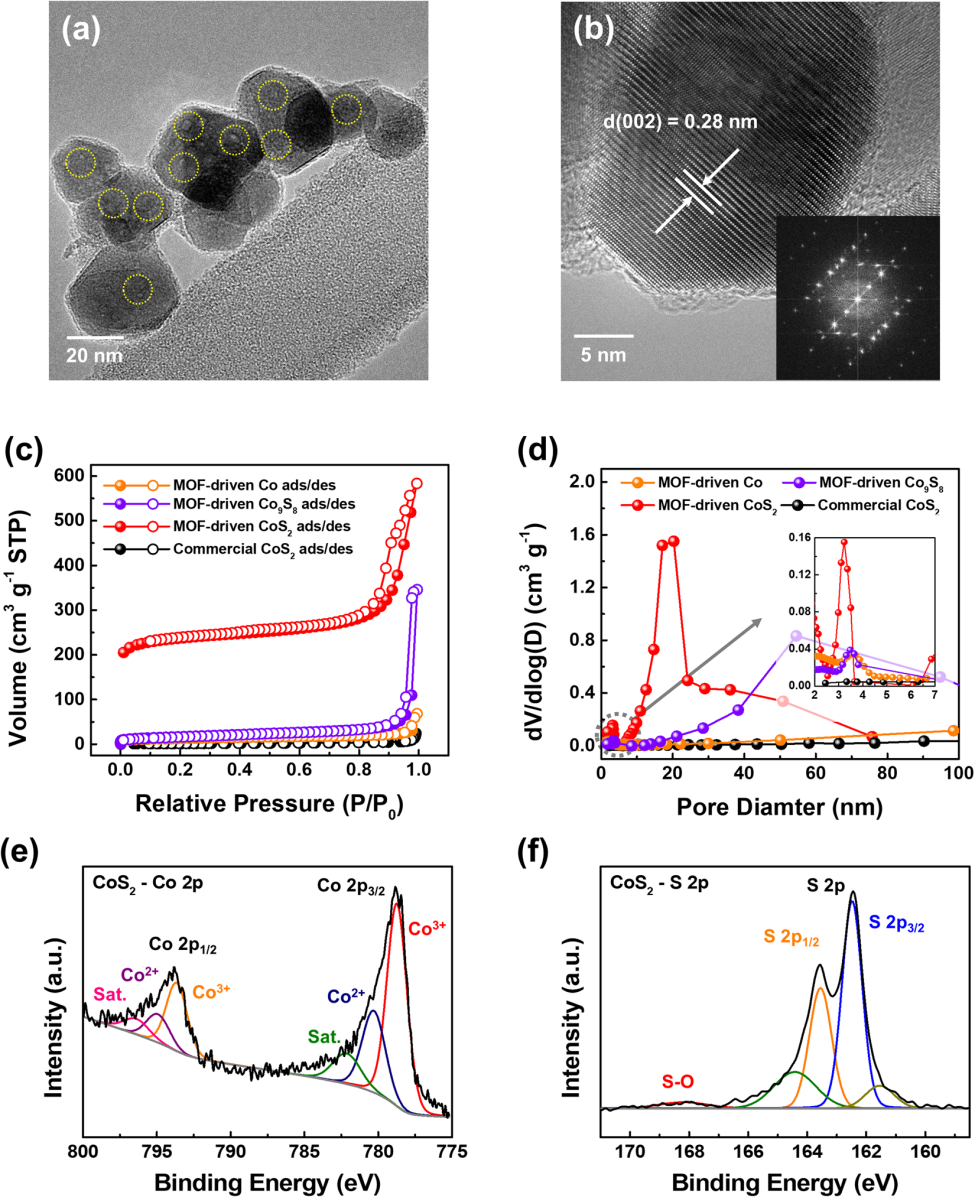
Microstructural and chemical characterization of MOF-driven porous CoS_2_ nanoparticles. (**a**) TEM image after sulfurization of Co-PBAs. (**b**) High-resolution TEM image and fast Fourier transformation (FFT) analysis (inset) of MOF-driven CoS_2_. Brunauer-Emmett-Teller (BET) analysis of (**c**) isothermal plot with N_2_ adsorption/desorption and (**d**) pore diameter distribution of MOF-driven Co, Co_9_S_8_, CoS_2_, and commercial CoS_2_ (inset: magnification of the small pore diameter area). (**e**,**f**) X-ray photoelectron spectroscopy (XPS) analysis for observation of bonding of CoS_2_. (**e**) Cobalt 2p spectrum and (**f**) sulfur 2p spectrum of MOF-driven CoS_2_.

Figure 2 shows the results of microstructural, chemical, and spectroscopic analyses to characterize MOF-driven CoS_2_ nanoparticles. The SEM (Fig. S4a) and TEM images (Fig. 2a) after sulfurization show that the MOF-driven porous CoS_2_ had uniform particle sizes of approximately 25 nm. Using high-resolution TEM, as shown in Fig. 2b, it was confirmed that the MOF-driven CoS_2_ had uniform crystallinity for each nanoparticle with a *d*-spacing of 0.28 nm, which corresponds to the (002) planes. The same result was obtained from the FFT pattern in the inset of Fig. 2b, which revealed that the MOF-driven CoS_2_ was highly crystalline with an (002) preferred orientation. The energy dispersive X-ray spectroscopy (EDX) was shown that the Co and S of synthesized MOF-driven CoS_2_ were uniformly distributed (Fig. S4b). Microstructural characterization of MOF-driven Co, Co_9_S_8_ (0, 0.1 ratio of sulfur/Co-PBAs), and commercial CoS_2_ were displayed in Fig. S5. It is confirmed that the higher S/Co ratio, the smaller the particle size. The electron energy loss spectroscopy (EELS) was conducted due to verification of charge distribution of MOF-driven cobalt compounds (Fig. S6). The chemical shift of Co L_23_ edge to higher energy was observed depending on higher S/Co ratio of cobalt sulfide^42^.

To further verify the formation of porous structures in the MOF-driven CoS_2_, shown in Fig. 2c,d, BET analysis was completed, where the BET specific surface area and pore diameter were obtained. In the case of MOF-driven CoS_2_, the BET specific surface area in Fig. 2c was 915.6 m^2^ g^−1^, compared with 6.1, 46.0, and 60.1 m^2^ g^−1^ for commercial CoS_2_, MOF-driven Co, and Co_9_S_8_, respectively (Co-PBAs: 43.2 m^2^ g^−1^ in Fig. S7). This result implies that MOF-driven CoS_2_ had 150 times higher porosity than that of commercial CoS_2_. Furthermore, the Barrett-Joyner-Halenda (BJH) desorption was measured to quantitatively express the pore diameter shown in Fig. 2d. Two main peaks were observed in the BJH results, and the peaks appeared at approximately 3.5 nm in MOF-driven CoS_2_ (red line) and at ~20 nm (gradually increasing from 6 nm). A gradually increasing peak includes the surface of particles because there is no selectivity (pore and surface) for the adsorption/desorption of N_2_ during BET measurements. Although relatively low intensity, the peak of MOF-driven Co, Co_9_S_8_ were also detected at about 3.5 nm. Increasing the sulfur ratio leads to increasing the sulfur base gas (Fig. S3), which can be related to change of particle size and pore distribution. However, the pore size was not nearly detected in the commercial CoS_2_.

The porous structures were also observed in the TEM images, which confirmed the BET analysis. In Fig. 2a, there are bright parts in the TEM image that are highlighted with dotted yellow circles^43–46^. As shown in the TEM image, the size of the bright area was approximately 3 to 4 nm. Additionally, the results of electrochemical surface area (ECSA) were shown in Fig. S8. The linear slopes of commercial CoS_2_ on Ni foam electrode and MOF-driven CoS_2_ on Ni foam electrode are 0.7 and 1.5 mF cm^−2^ respectively, confirmed that the ECSA difference was about 2.1-fold (MOF-driven Co: 0.7, Co_9_S_8_: 1.1 mF cm^−2^). The ECSA of each electrode was calculated in Fig. S8.

XPS was conducted to observe the chemical and electronic states of MOF-driven CoS_2_ as shown in Fig. 2e,f. The broad scan in Fig. S9a shows peaks at binding energies of approximately 780, 530, 400, 286, and 163 eV, which were indexed to Co 2p, O 1 s, N 1 s, C 1 s, and S 2p, respectively. Specifically, in Fig. 2e, the Co 2p spectrum consists of peaks at 794 and 779 eV, which correspond to Co 2p_1/2_ and Co 2p_3/2_ respectively. Each peak can split into three sub-peaks, which are located at 796.7, 795.1 and 793.7 eV for the Co 2p_1/2_ spectrum and 781, 779.7 and 778.5 eV for the Co 2p_3/2_ spectrum. As indicated in Fig. 2e, the sub-peaks are known as satellites corresponding to Co^2+^ and Co^3+^ ^6,10,26,47–49^. The S 2p spectrum shown in Fig. 2f can typically be split two sub-parts with peaks at 168 eV and 163 eV. The 163 eV peak generally indicates the ‘−1’ valence state of sulfur, with sub-peaks indexed as 2p_1/2_ (163.6 eV) and 2p_3/2_ (162.5 eV). The peak at 168 eV is typically indexed as a sulfur oxide^32,50^. In this result, the sulfur oxide peak has a negligibly low intensity compared with other previous reports that describe hydrothermally synthesized CoS_2_. The results of the physical and chemical property analysis through XRD and XPS show that the synthesized CoS_2_ had high purity.

### Investigation of electrochemical characterization

**Figure 3:**
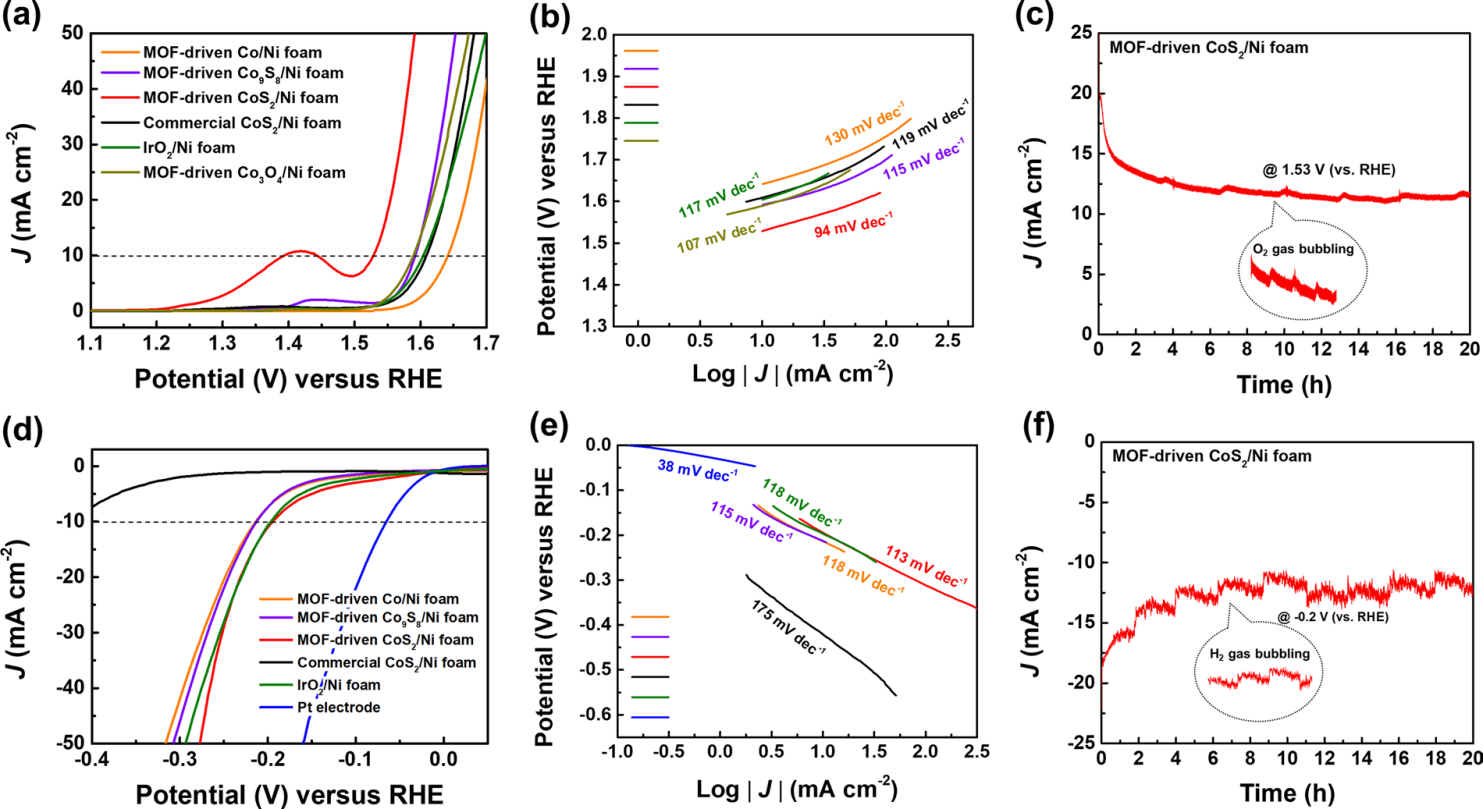
Electrochemical characterization of MOF-driven porous CoS_2_ nanoparticles in 1.0 M KOH. (**a**) OER polarization curves of electrodes with a scan rate of 1 mV s^−1^. (**b**) Tafel slopes of polarization curves in OER. (**c**) Chronoamperometric stability test of fabricated CoS_2_ at constant voltage. (**d**) HER polarization curves of electrodes with a scan rate of 5 mV s^−1^. (**e**) Tafel slopes of polarization curves in HER. (**f**) Chronoamperometric stability test of fabricated CoS_2_ at constant voltage.

As shown in Fig. 3, to examine the bifunctional electrocatalytic activity of the MOF-driven porous CoS_2_ nanoparticles on the Ni foam electrode, linear sweep voltammetry (LSV) was conducted for the bare Ni foam, Ir foil, Pt electrode, commercial CoS_2_, and MOF-driven CoS_2_. The OER and HER were performed with a conventional three-electrode system in 1.0 M KOH (see for methods section for details). Polarization measurements were conducted in the potential range between 1.2 and 1.7 V (vs. reversible hydrogen electrode (RHE)) for the OER at a scan rate of 1 mV s^−1^ and between −0.4 and 0.0 V (vs. RHE) for the HER at a scan rate of 5 mV s^−1^. First, the OER activity of the electrocatalysts was measured as the overpotential at a current density of 10 mA cm^−2^ and the Tafel slope. Figure 3a displays the polarization curves of the OER range. The MOF-driven CoS_2_ electrode shows a low overpotential requirement of 298 mV at 10 mA cm^−2^, while the others show a higher overpotential. Under the same conditions, the overpotentials of the MOF-driven Co, MOF-driven Co_9_S_8_, commercial CoS_2_, IrO_2_, and MOF-driven Co_3_O_4_ were 411, 362, 379, 374, and 359 mV, respectively. The LSVs of Ir foil, Pt electrode, and Ni foam were conducted (Fig. S10a). The activity of MOF-driven Co_3_O_4_ was investigated for comparison with MOF-driven CoS_2_. This Co_3_O_4_ was fabricated with annealed Co-PBAs at same temperature condition in air, which was confirmed by XRD (Fig. S11).

Additionally, the oxidation peak in the polarization curve was confirmed to be from the Co(III) to Co(IV) transition^51^. The MOF-driven CoS_2_ shows a larger oxidation peak in this location than that in previously reported studies. The larger the specific surface area, the greater the intensity of the redox peak.

The Tafel plot was plotted based on the polarization curves, and the Tafel slope was calculated (Fig. 3b). As with many other reported results, the tendency of the Tafel slope and overpotential were similar. The synthesized CoS_2_ electrode, which had the lowest overpotential in the polarization curves, showed a low Tafel slope value (94 mV dec^−1^).

In Fig. S12, electrochemical impedance spectroscopy (EIS) of commercial CoS_2_, 0, 0.1, and 0.5 ratios of sulfur/Co-PBAs (MOF-driven Co, Co_9_S_8_, and CoS_2_, respectively) was conducted at 300 mV (vs. RHE). Equivalent circuit model was shown in inset of Fig. S12. According to the EIS result, MOF-driven CoS_2_ was shown the lowest charge transfer resistance.

To verify the stability of the MOF-driven porous CoS_2_ nanoparticles, a long-term durability test was conducted using the chronoamperometric method (Fig. 3c). At a constant voltage of 1.53 V (vs. RHE), the synthesized CoS_2_ electrode delivered a relatively stable current density of 12 mA cm^−2^ over 20 hours. As oxygen evolution occurs during the test, fluctuations were observed due to gas bubbling.

Next, HER electrocatalytic activity was assessed as the overpotential at a current density of 10 mA cm^−2^ and the Tafel slope. As shown Fig. 3d, the HER overpotential was confirmed through LSV. The MOF-driven CoS_2_ electrode displays a low overpotential requirement of −196 mV at 10 mA cm^−2^, while the commercial CoS_2_ shows a large overpotential. The overpotentials of the MOF-driven Co, MOF-driven Co_9_S_8_, IrO_2_, and Pt electrode are −214, −213, −198, and −67 mV, respectively. The LSVs of Ir foil, Ni foam were conducted (Fig. S10c). The synthesized CoS_2_ electrode also shows high activity in the HER despite the presence of a strong alkaline electrolyte.

As shown in Fig. 3e, the Tafel slope was calculated based on the HER polarization curves, as in the OER results. Under the overpotential results, Pt and synthesized CoS_2_ electrodes exhibit Tafel slopes of 38 and 113 mV dec^−1^, respectively. Therefore, the synthesized CoS_2_ electrode shows the Volmer-Heyrovsky mechanism behavior^52^.

To confirm the stability in HER activity, a long-term durability test was also conducted with chronoamperometry (Fig. 3f). At a constant voltage of −0.2 V (vs. RHE), the synthesized CoS_2_ electrode delivered a relatively stable current density of −11 mA cm^−2^ over 20 hours. As hydrogen evolution occurs during the test, fluctuations were also observed due to gas bubbling.

**Figure 4:**
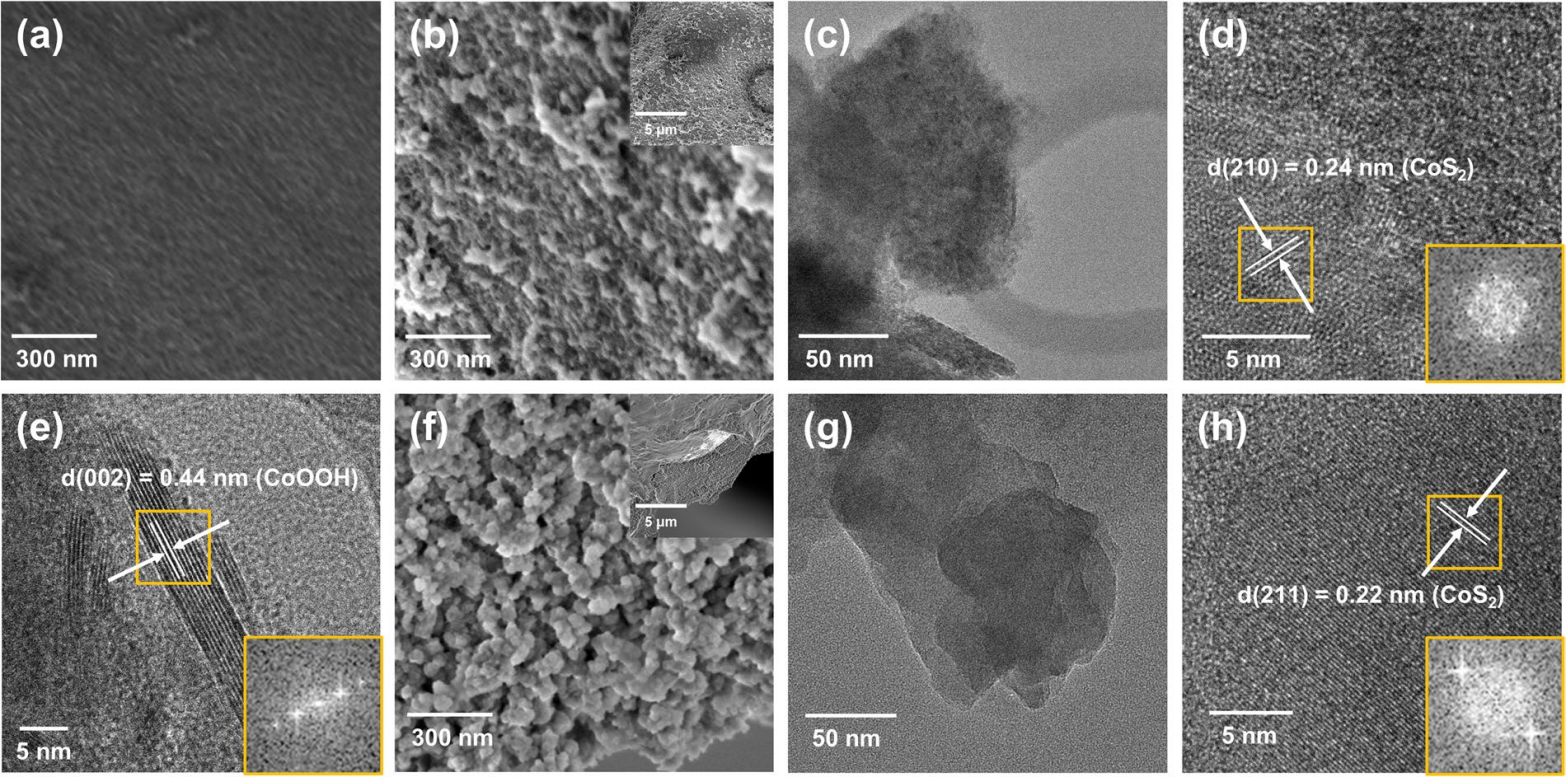
Microstructural characterization of MOF-driven CoS_2_ electrode after durability test. (**a**) FE-SEM image of the bare Ni foam. (**b**–**e**) Electron microscopy images of MOF-driven CoS_2_ electrode after OER durability at 1.53 V (vs. RHE). (**b**) FE-SEM (inset: low magnification) (**c**) TEM, and (**d**,**e**) high-resolution TEM (inset: FFT analysis) images. (**f**,**g**) Electron microscopy images of MOF-driven CoS_2_ electrode after HER durability at −0.2 V (vs. RHE). (**f**) FE-SEM (inset: low magnification) (**g**) TEM, and (**h**) high-resolution TEM (inset: FFT analysis) images.

In Fig. 4, to further investigate the change of MOF-driven CoS_2_ upon electrochemical test, structural and chemical characterization was performed after OER and HER durability test. As shown in Fig. 4b,f, although slight change of morphology was shown in FE-SEM images after OER and HER durability test, the MOF-driven CoS_2_ nanoparticles well-retained on the Ni foam compared with Fig. 4a. This slight change is inevitable, since O_2_ and H_2_ gas was vigorously emitted during OER and HER durability test, respectively^10,53^. The XRD pattern of MOF-driven CoS_2_ electrode had hardly changed after HER, while peak of CoOOH was slightly detected in the electrode after OER (Fig. S13).

As shown in Fig. 4c–e, TEM analysis after OER durability was performed. The lattice fringes corresponding to CoS_2_ (Fig. 4d) and CoOOH (Fig. 4e) were observed. However, these lattice fringes featured low crystallinity, because of the crystalline to amorphous transition in electrocatalysts of transition metal compound^6^. EDX mapping after OER durability test of MOF-driven CoS_2_ electrode was conducted (Fig. S14a). Although the Co and O of MOF-driven CoS_2_ after OER durability test were uniformly distributed, signal of S was relatively weak due to the formation of surface CoOOH^54^. XPS analysis was conducted (Fig. S14b–d). The peak of CoOOH (783 eV) was observed in Co 2p spectrum^54,55^. In S 2p spectrum, only S-O peaks were featured^6,56^. The intensity of total O 1 s after OER was relatively increased compared to O 1 s before test. Furthermore, new peak was also observed at 529.9 eV (cobalt oxide)^51^.

After carrying out the HER durability test, TEM analysis was also conducted (Fig. 4f–h). Similar to the OER part, lattice fringe of low crystalline CoS_2_ was observed (Fig. 4h). EDX mapping and XPS analysis after HER test (Fig. S15). In EDX mapping, relatively weak signal of O and S was detected due to formation of transition chalcogenide hydrides during HER^10,57^. In XPS analysis, the peak of intensity at 781 eV (Co 2p_3/2_) was increased, which this phenomenon was also reported in other work^6^.

**Figure 5:**
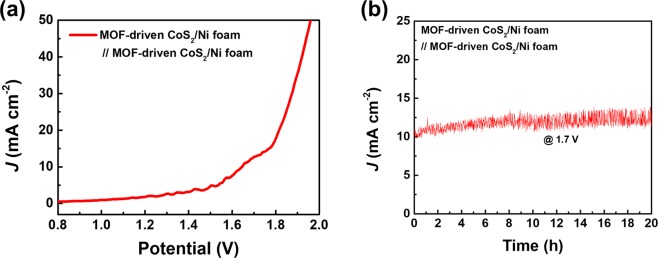
Overall water splitting performance of MOF-driven porous CoS_2_ nanoparticles. (**a**) Polarization curve and (**b**) chronoamperometric stability test of symmetric full cell.

Figure 5 shows the performance of bifunctional electrocatalytic activity, which was conducted through a full-cell test of electrochemical water splitting for a practical two-electrode system in 1.0 M KOH. Both the anode and cathode were assembled from the same electrode material, which was MOF-driven CoS_2_ on Ni foam. The potential range was from 0.0 to 2.0 V. As shown in Fig. 5a, the assembled full-cell exhibited an overpotential of 1.65 V at 10 mA cm^−2^. The durability test was also conducted for symmetric full-cells (Fig. 5b). At a constant voltage of 1.65 V, the synthesized CoS_2_ electrode delivered a relatively stable current density of 11 mA cm^−2^ over 20 hours.

As shown above, it was verified with structural characterization, chemical characterization and physical adsorption/desorption analysis that the synthesized CoS_2_ had a highly porous structure, including nanoscale pores. According to these results, the MOF-driven CoS_2_ nanoparticles provided facile diffusion kinetics as well as enough active sites. For this reason, the synthesized CoS_2_ electrode had a high OER and HER catalytic activities despite no additional treatment.

## Conclusion

Highly porous structures including nanoscale pores were effective at enhancing the OER and HER electrocatalytic activities of the mono-phase CoS_2_ nanoparticles and reached values of 298 and −196 mV at 10 mA cm^−2^, respectively. Thermodynamic calculations were performed for the predictive synthesis of CoS_2_ from a PBA, one of the MOFs. The structural characterization of the synthesized MOF-driven CoS_2_ confirmed the uniformity and porous structure. From the investigation of the electrochemical characterization of the MOF-driven CoS_2_ electrode, the electrocatalytic performance of water splitting was high due to the kinetically favorable porous structure. Furthermore, the possibility of a bifunctional electrocatalyst was confirmed with a full-cell system.

## Methods

### Preparation of porous CoS_2_ nanoparticles

First, the cobalt Prussian blue analogue (Co-PBA), namely Co_3_[Co(CN)_6_]_2_ powder, was used as a starting material before sulfurization. The Co-PBA was synthesized through a facile precipitation method. Two types of solutions were used for precipitation. The recipe for the type 1 solution was 30 mM cobalt acetate (Co(CH_3_COO)_2_∙4H_2_O, Aldrich) and 17 mM sodium citrate (Na_3_C_6_H_5_O_7_∙2H_2_O, Sigma-Aldrich) in deionized water (DIW). The concentration of potassium hexacyanocobaltate (III) (K_3_[Co(CN)_6_], Sigma-Aldrich) was 20 mM in DIW^41^. Then, the type 1 solution was dropwise added into the type 2 solution through a syringe pump at 400 ml/hr and vigorously stirred for 10 min. Subsequently, the mixed solution was aged for 18 hours at room temperature. The dispersed precipitate was filtered through a vacuum pump and washed with DIW several times to remove residues. Finally, the obtained powder was dried in an oven at 60 °C for 12 hours.

Second, the cobalt disulfide (CoS_2_) nanoparticles were synthesized through thermal treatment. The 30 mg of the synthesized Co-PBA and certain amounts of sulfur (S, Sigma-Aldrich) were loaded in a 5 ml ampoule. After that, the prepared ampoule was sealed through a vacuum pump under 0.1 Torr. Finally, the sealed ampoule was thermally treated in a furnace at 500 °C for 2 hours at a ramp rate of 5 °C/min^7^.

### Preparation of catalyst samples

To estimate the electrochemical performance, an ink was prepared. The 4 mg of synthesized CoS_2_ nanoparticles were dispersed in a mixture of DIW (800 μl), ethanol (200 μl, Sigma-Aldrich), and Nafion (80 μl, Aldrich) and sonicated for 30 min. After dispersion, 50 μl of the mixed ink was coated on the Ni foam (Aldrich) by drop casting through a micropipette. Finally, the coated electrode was dried in an oven at 60 °C for 12 hours. The ink preparation for the Co, Co_9_S_8_, Co_3_O_4_, commercial CoS_2_ powder (Aldrich), and IrO_2_ powder (Aldrich) was similar.

### Thermodynamic calculation

The ternary phase diagram, and analysis graphs of expected products depending on sulfur amount were drawn with a thermochemical database program (FACTSAGE software). The database used in this study was the FACT pure substances database (FactPS)^58^.

### Microstructural characterization

Phase analysis of the synthesized nanoparticles was performed using XRD (New D8 Advance, Bruker). Microstructural analysis of the synthesized CoS_2_ nanoparticles, including the Co-PBA, and acquisition of the FFT patterns was conducted using TEM (JEM-2100F, JEOL Ltd.). Additionally, the microstructure, including the size and morphologies of the synthesized nanoparticles, was observed using FE-SEM (SIGMA, Carl Zeiss). The surface area was conducted using a BET analyzer with N_2_ adsorption/desorption at 77.3 K (ASAP2420, Micromeritics Instruments).

### Electrochemical characterization

LSV of each electrode was performed in a three-electrode system using a potentiostat (ZIVE MP2A, Wonatech, Korea). The working electrode was the prepared catalyst electrode. The counter and reference electrodes were Pt foil and a saturated calomel electrode (SCE) (Qrins, Korea), respectively, in 1.0 M KOH (Sigma-Aldrich). The LSV was conducted in the potential range between 1.2 and 1.7 V (vs. RHE) with a scan rate of 1 mV s^-1^ for the OER and between −0.4 and 0.0 V (vs. RHE) for the HER. In the case of overall water splitting, both electrodes were prepared using a potential range between 0.0 and 2.0 V. In HER and overall water splitting LSVs, the scan rate was fixed at 5 mV s^−1^. Electrochemical surface area (ECSA) was conducted using cyclic voltammetry (CV). CV was conducted in the voltage range between 0.18 and 0.28 V (vs. RHE) at a scan rate of 5, 10, 25, 50, 100, 200, 400, 600, 800, and 1000 mV s^−1^. The linear slope was calculated by plotting Δ*J* at 0.23 V (vs. RHE) depending on scan rates^59^. EIS was performed within a frequency range from 100,000 Hz to 0.01 Hz at 300 mV (vs. RHE)^60^.

## Supplementary information

Supplementary Information

## Data Availability

The data that support the findings of this study are available from the corresponding author on reasonable request.
