# Supplementary material for: Metal-organic Framework-driven Porous Cobalt Disulfide Nanoparticles Fabricated by Gaseous Sulfurization as Bifunctional Electrocatalysts for Overall Water Splitting
Source: Sci Rep. 2019 Dec 20;9:19539. doi: 10.1038/s41598-019-56084-9 (PMC6925291; doi:10.1038/s41598-019-56084-9)
Supplement: Supplementary file 1 — Supplementary Information [file 41598_2019_56084_MOESM1_ESM.docx]

Supplementary Information for

Metal-Organic Framework-driven Porous Cobalt Disulfide Nanoparticles Fabricated by Gaseous Sulfurization as Bifunctional Electrocatalysts for Overall Water Splitting

In-Kyoung Ahn^1^, Wonhyo Joo^1^, Ji-Hoon Lee^2^, Hyoung Gyun Kim^1^, So-Yeon Lee^1^, Youngran Jung^1^, Ji-Yong Kim^1^, Gi-Baek Lee^1^, Miyoung Kim^1^, & Young-Chang Joo^1,3,^*

*^1^Department of Materials Science & Engineering, Seoul National University, Seoul 08826, Republic of Korea*

*^2^Materials Center for Energy Convergence, Surface Technology Division, Korea Institute of Materials Science (KIMS), Changwon, Gyeongnam 51508, Republic of Korea*

*^3^Research Institute of Advanced Materials (RIAM), Seoul National University, Seoul 08826, Republic of Korea*

**Corresponding Authors**

Young-Chang Joo (ycjoo@snu.ac.kr, Tel.: +82-2-880-8986)

**
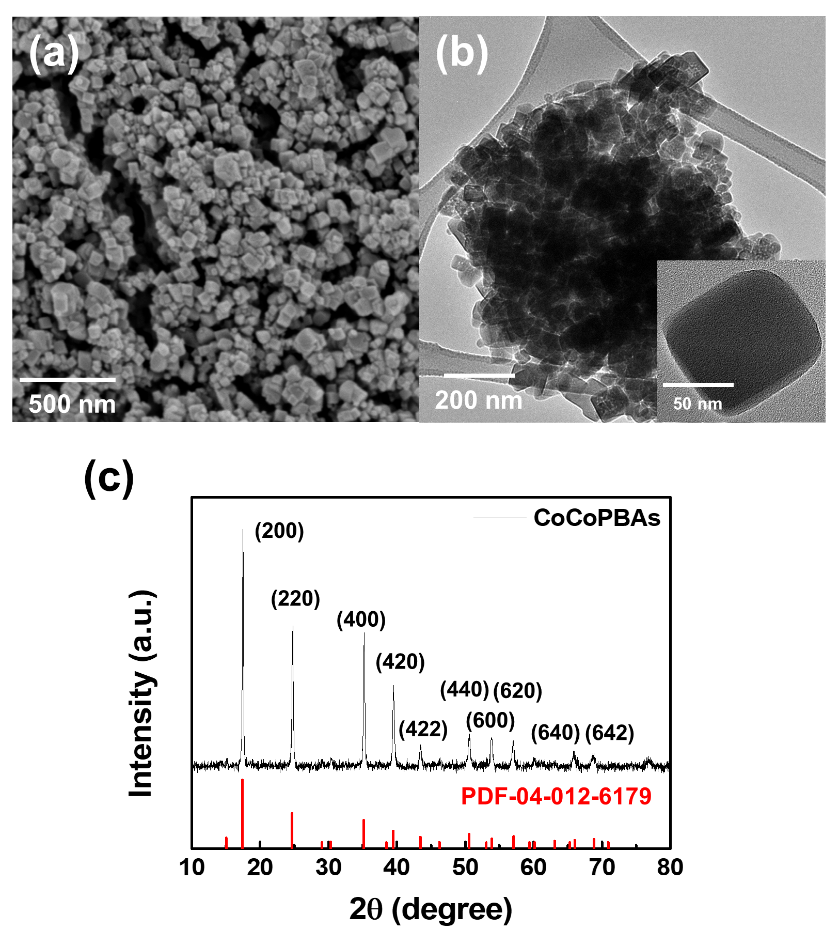
**

**Figure S1.** Characterization of Co-PBA nanoparticles. (a) FE-SEM and (b) TEM images after synthesis of Co-based MOF. (c) XRD analysis for verification of Co-PBA.

**
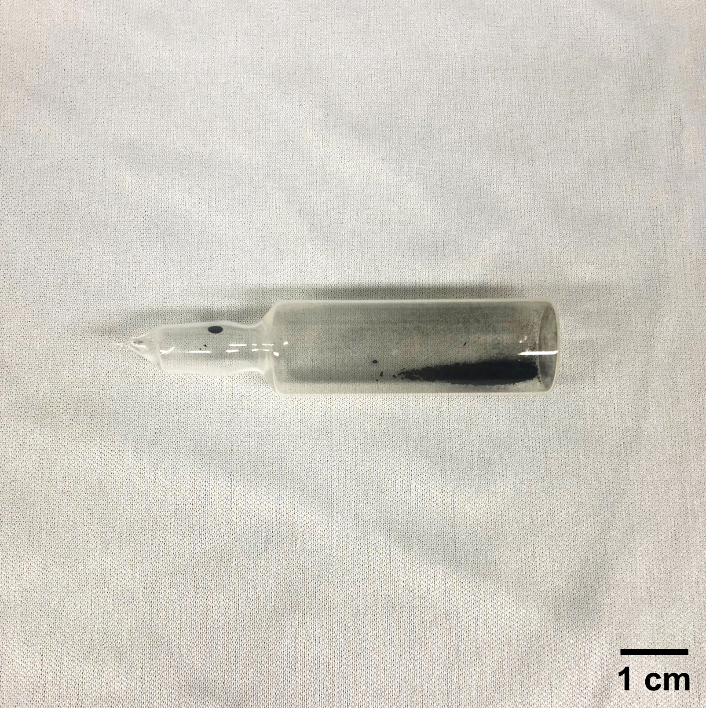
**

**Figure S2.** Image of sealed glass ampoule. Co-PBA and sulfur powder were inserted, and the pressure inside was maintained 0.1 Torr.

**
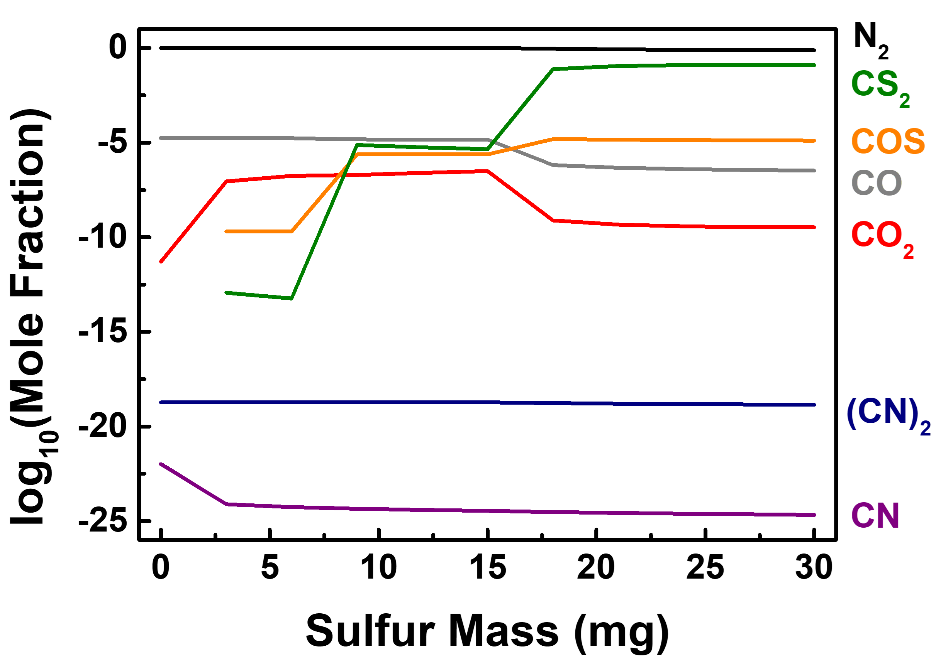
**

**Figure S3.** Sulfurization of Co-PBA related gaseous decomposition products. In sulfurization temperature (500 °C), other products were completely vaporized except for the CoS_2_, target phase.

**
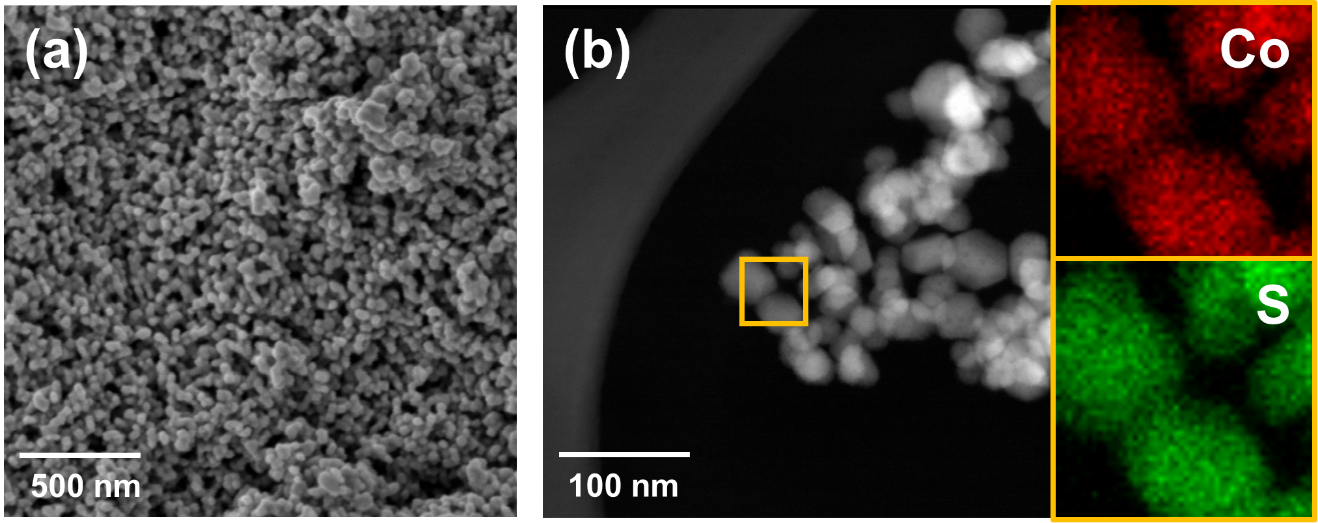
**

**Figure S4** Characterization of MOF-driven CoS_2_. (a) FE-SEM image. (b) STEM image and EDX elemental mapping images (red: Co, green: S in inset).

**
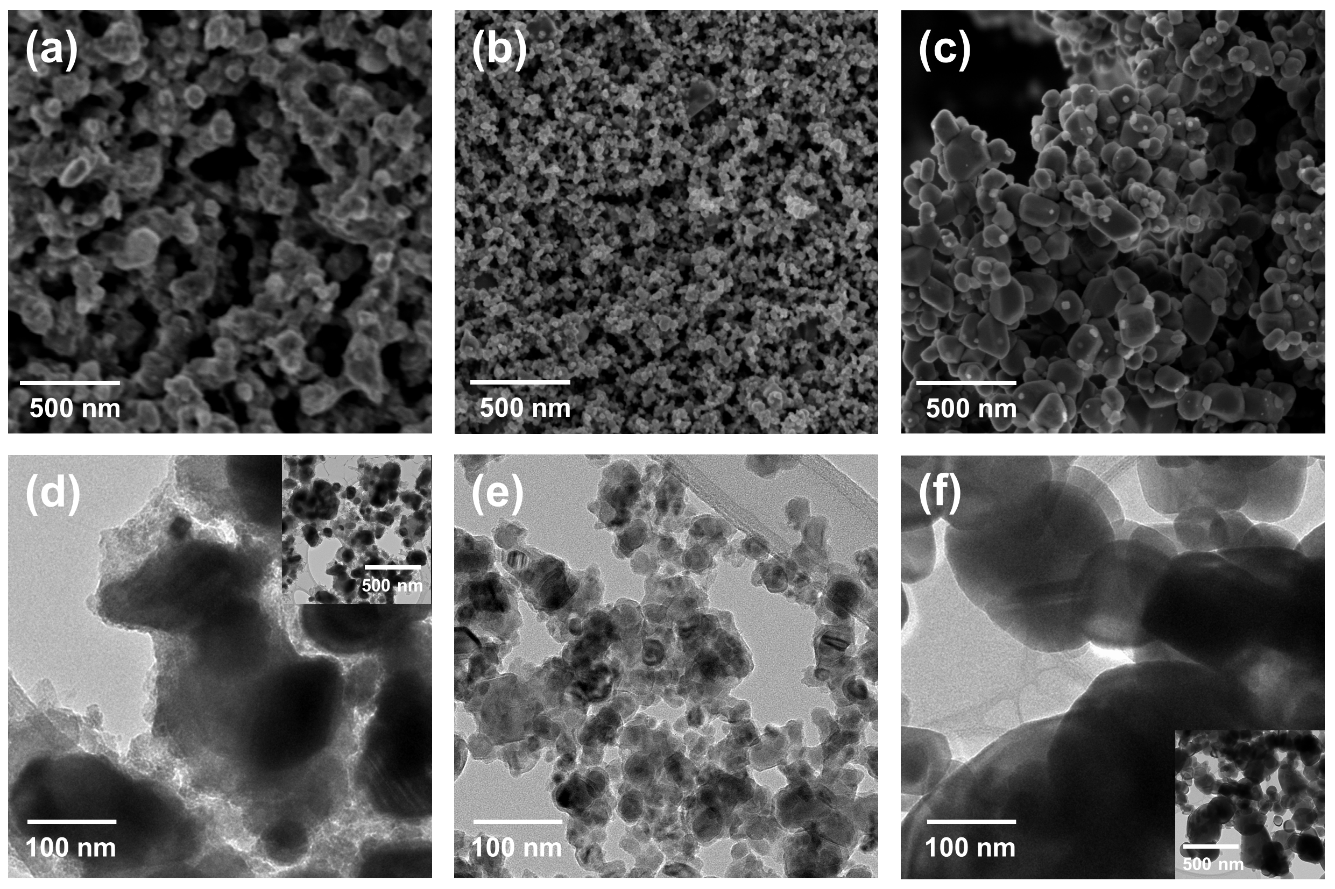
**

**Figure S5.** Microstructural characterization of sulfurized Co-PBAs (MOF-driven Co, Co_9_S_8_) and commercial CoS_2_. (a-c) FE-SEM images and (d-f) TEM images of each cobalt compound, (a), (d) MOF-driven Co, (b), (e) MOF-driven Co_9_S_8_, and (c), (f) commercial CoS_2_.

**
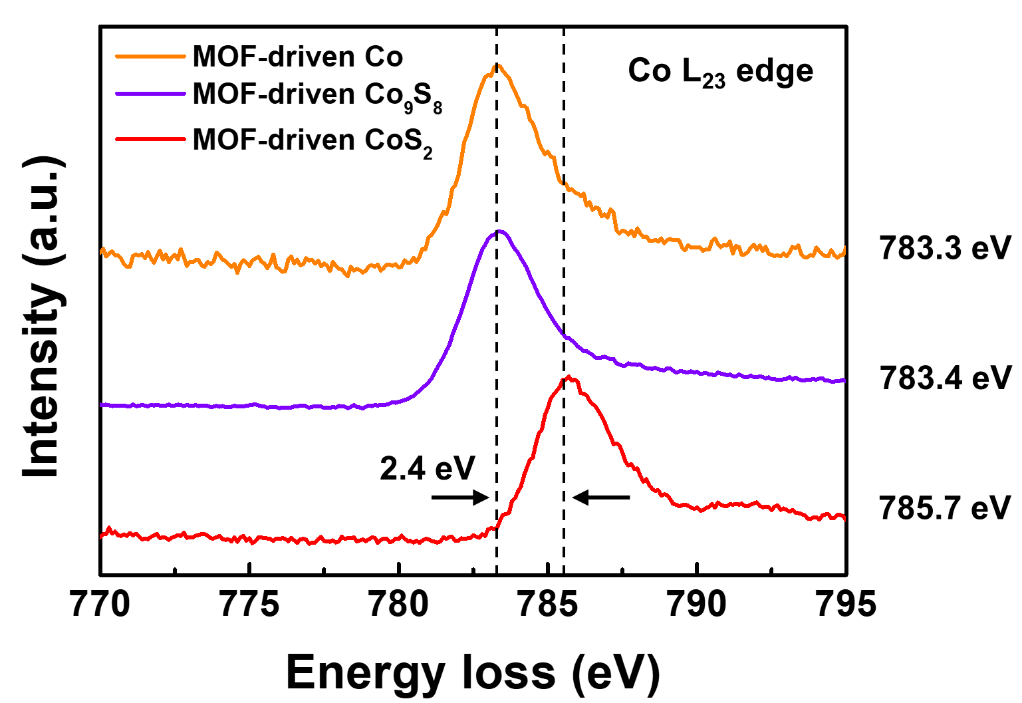
**

**Figure S6.** EELS spectrum analysis of Co L_23_ edge for verification of sulfurization from MOF-driven Co, Co_9_S_8_, and CoS_2_ (S/Co-PBAs weight ratio: 0, 0.1, and 0.5).

**
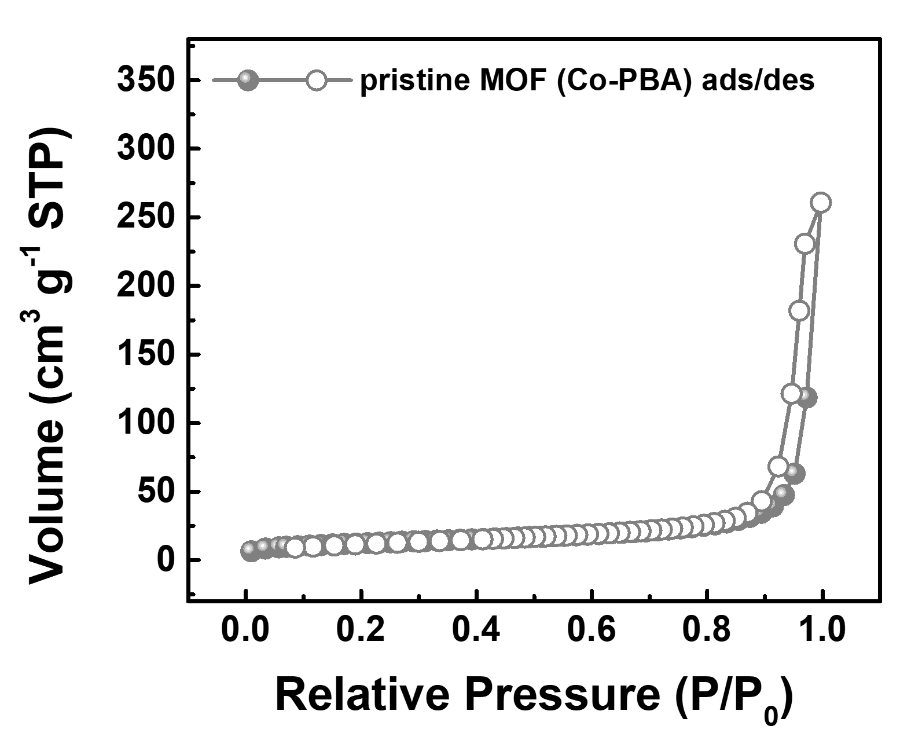
**

**Figure S7.** BET analysis of isothermal plot with N_2_ adsorption/desorption in pristine MOF (43.2 m^2^ g^-1^, before sulfurization)


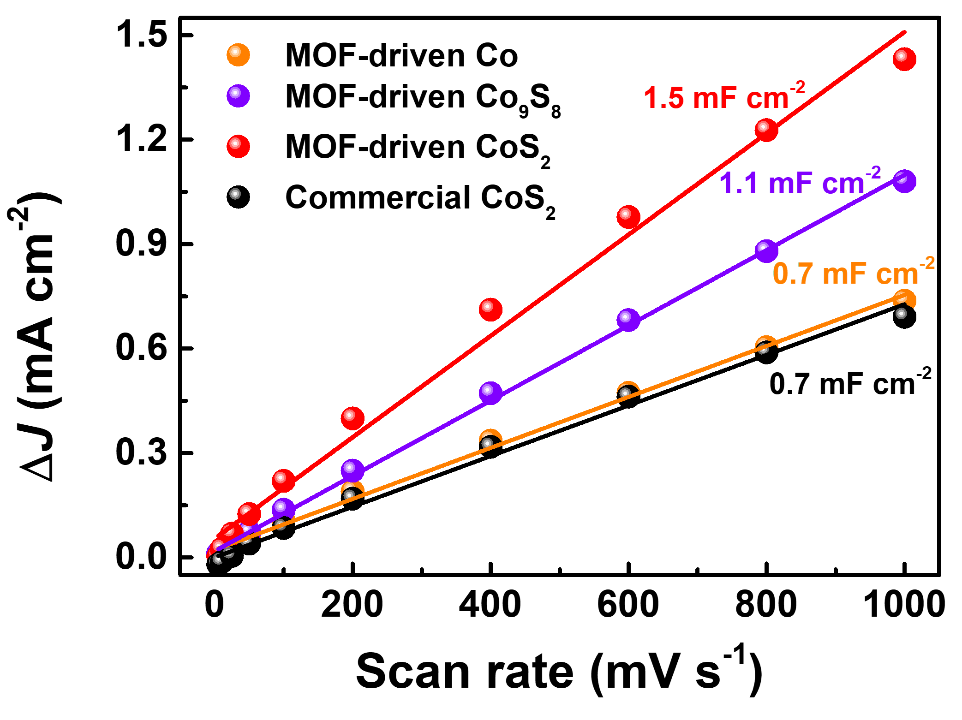


**Figure S8.** ECSA of commercial CoS_2_, MOF-driven Co, Co_9_S_8_, and CoS_2_ (S/Co-PBAs weight ratio: 0, 0.1, and 0.5). The linear slope is related to the double layer capacitance.

CV was conducted in the voltage range between 0.18 and 0.28 V (vs. RHE). The linear slope was calculated by plotting Δ*J* at 0.23 V (vs. RHE) depending on scan rates. The linear slope is two-fold of the double-layer capacitance (C_dl_)^S1,S2^.

Δ*J* = (*J_a_ - J_c_*), C_dl_ can be calculaged following equation:

C_dl_ = 1/2 x d(Δ*J*)/d*ν*

The ECSA can be calculated following equation^S3-S5^:

ECSA = C_dl_/C_s_, C_s_: The capacitance of atomically smooth planar surface in 1.0 M KOH electrolyte ranges between 20 to 60 μF cm^-2 S3-S5^. For calculation, C_s_ was configured at 40 μF cm^-2 S3,S5^.

| **Electrocatalysts** | **Slope** | **C_dl_** | **ECSA** |
| --- | --- | --- | --- |
| MOF-driven Co/Ni foam | 0.7 mF cm^-2^ | 0.35 mF cm^-2^ | 8.75 cm^2^ _ECSA_ |
| MOF-driven Co_9_S_8_/Ni foam | 1.1 mF cm^-2^ | 0.55 mF cm^-2^ | 13.75 cm^2^ _ECSA_ |
| MOF-driven CoS_2_/Ni foam | 1.5 mF cm^-2^ | 0.75 mF cm^-2^ | 18.75 cm^2^ _ECSA_ |
| Commercial CoS_2_/Ni foam | 0.7 mF cm^-2^ | 0.35 mF cm^-2^ | 8.75 cm^2^ _ECSA_ |

**
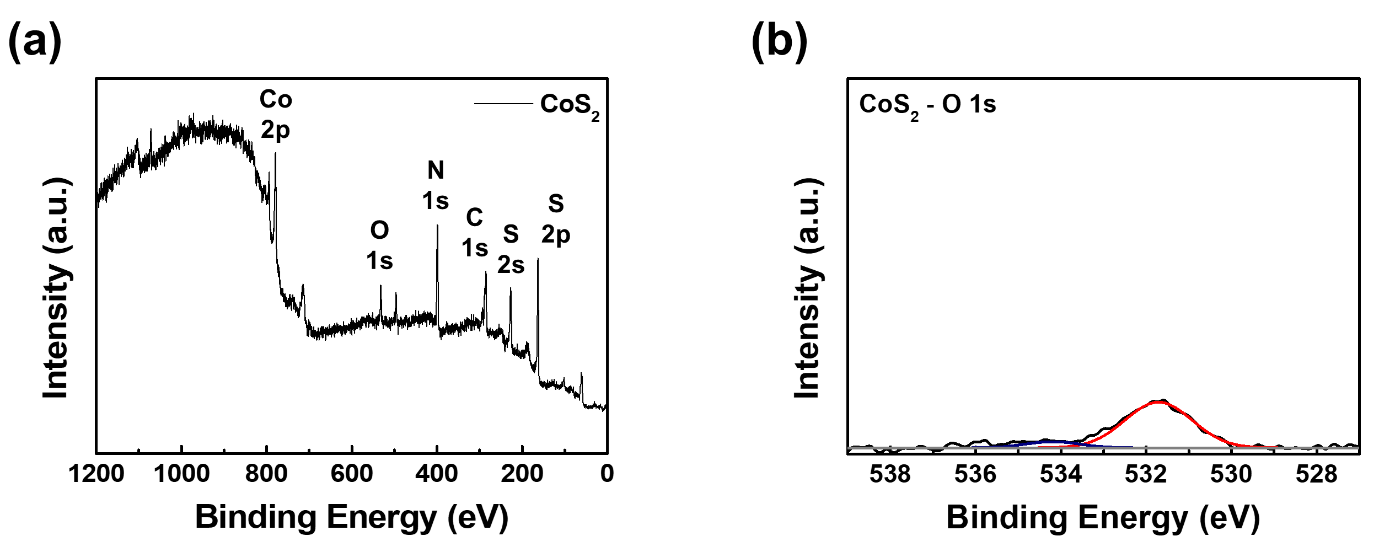
**

**Figure S9.** XPS analysis. (a) The broad scan of CoS_2_ nanoparticles. The peaks at binding energies of approximately 780, 530, 400, 286, and 163 eV, which were indexed to Co 2p, O 1s, N 1s, C 1s, and S 2p, respectively. (b) O 1s spectrum.

**
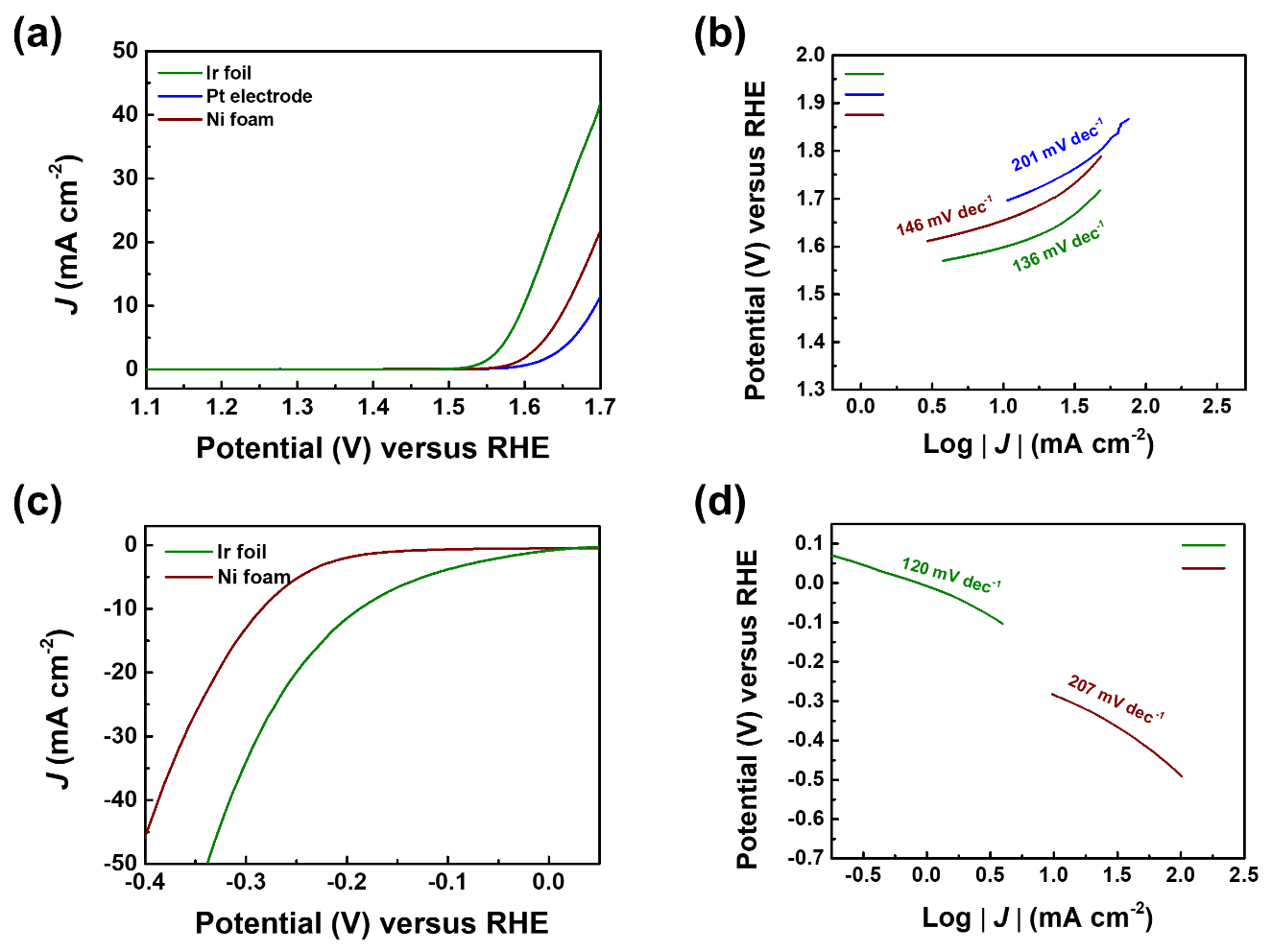
**

**Figure S10.** Electrochemical characterization of Ir foil, Ni foam, and Pt electrode. (a) OER polarization curves. (b) Tafel slopes of polarization curves in OER. (c) HER polarization curves. (d) Tafel slopes of HER.

**
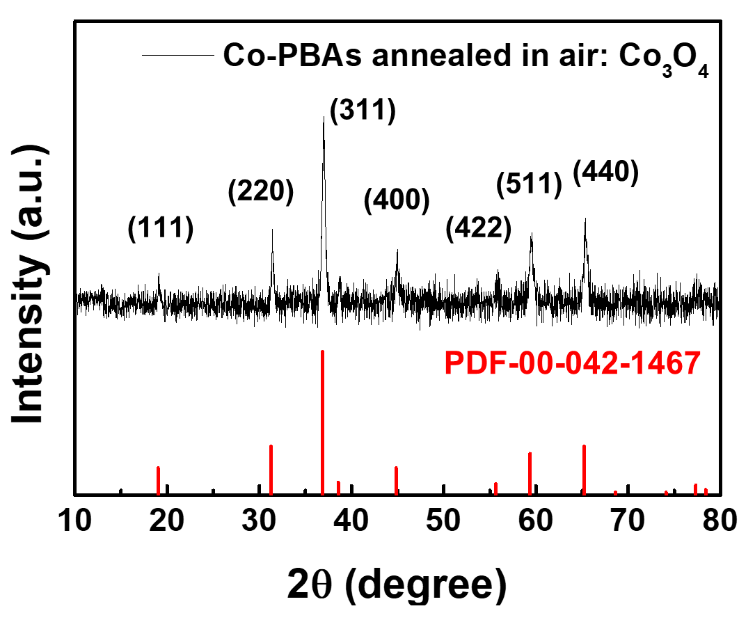
**

**Figure S11.** XRD analysis of annealed Co-PBAs in 500 °C, air.


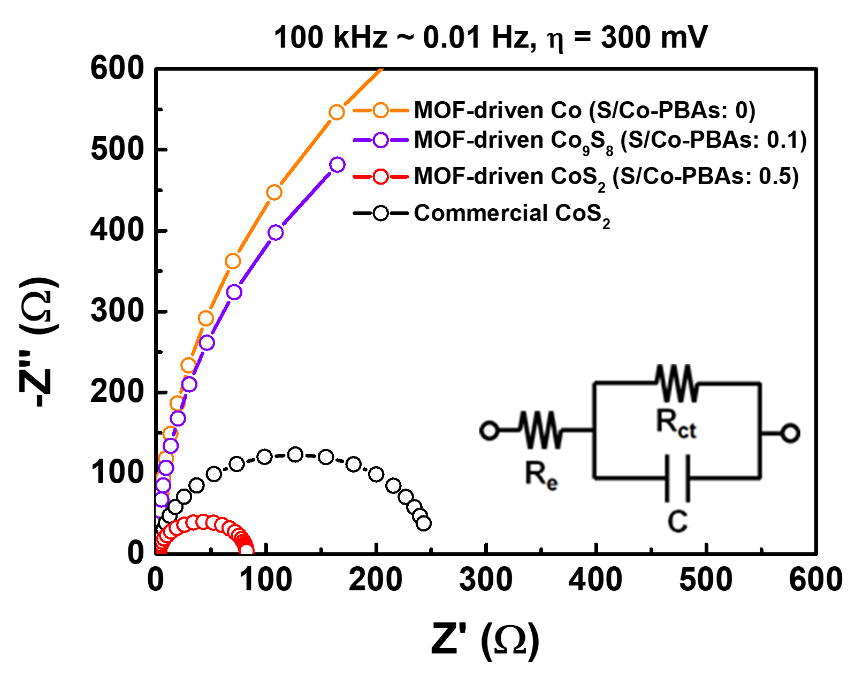


**Figure S12.** Nyquist plots at 300 mV (vs. RHE) for commercial CoS_2_, MOF-driven Co, Co_9_S_8_, and CoS_2_ (S/Co-PBAs weight ratio: 0, 0.1, and 0.5) electrodes. Inset is equivalent circuit model.


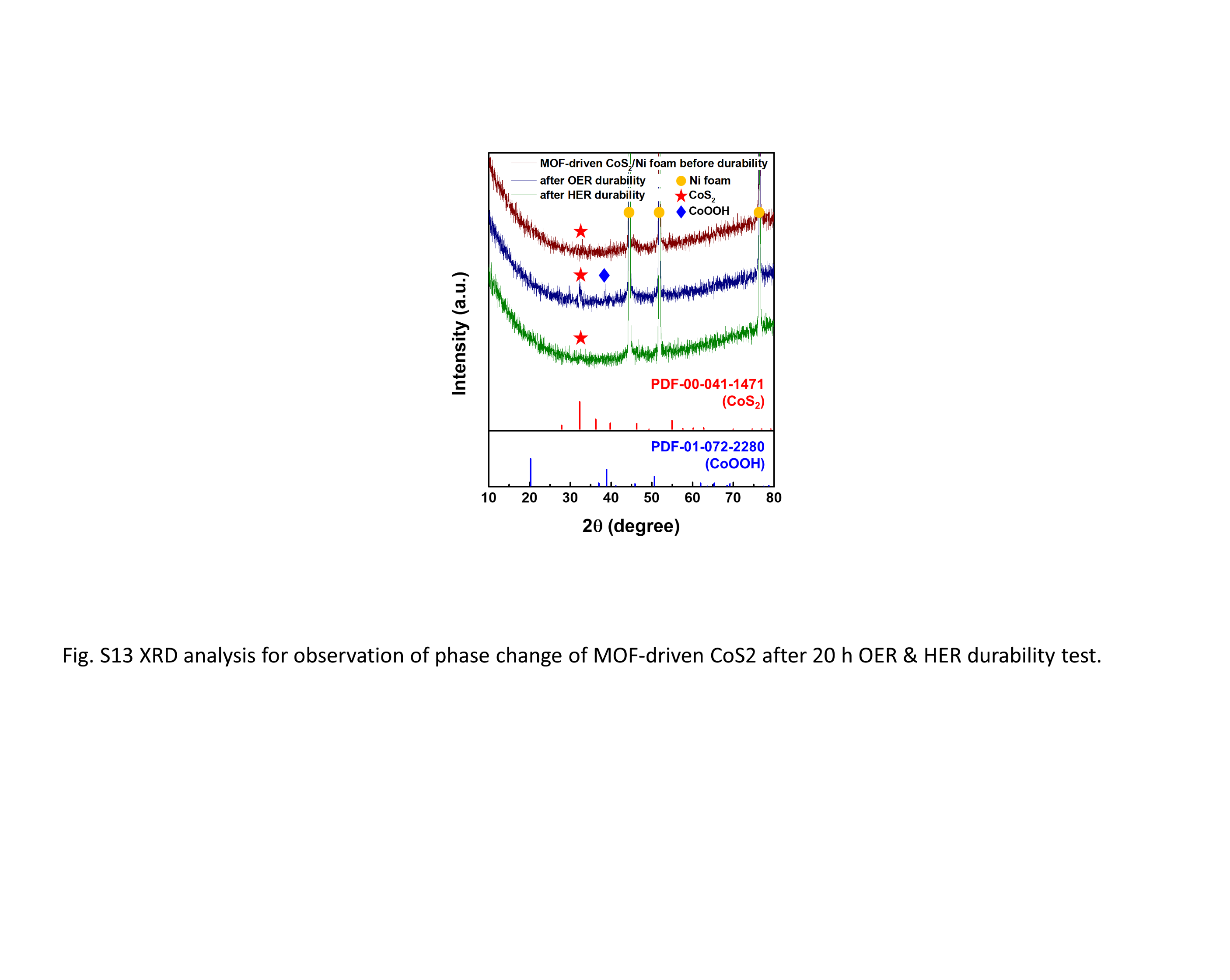


**Figure S13.** XRD analysis for observation of phase change of MOF-driven CoS_2_ after 20 h OER & HER durability test.


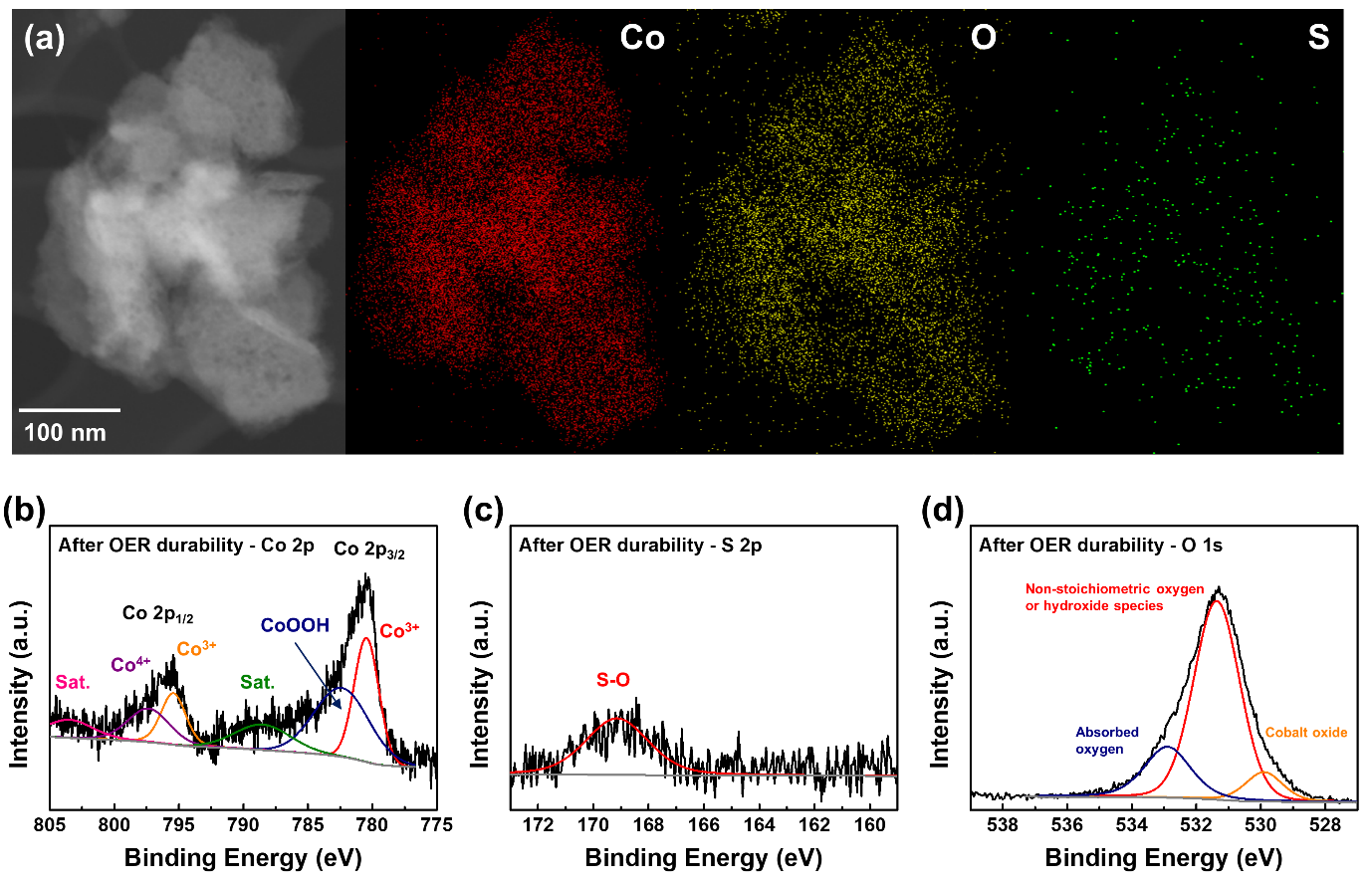


**Figure S14.** Chemical composition and state change of MOF-driven CoS_2_ after 20 h OER durability at 1.53 V (vs. RHE). (a) STEM image and EDX elemental mapping images (red: Co, yellow: O, green: S in inset). (b-d) XPS analysis. (b) Cobalt 2p, (c) sulfur 2p, and (d) oxygen 1s spectrum.


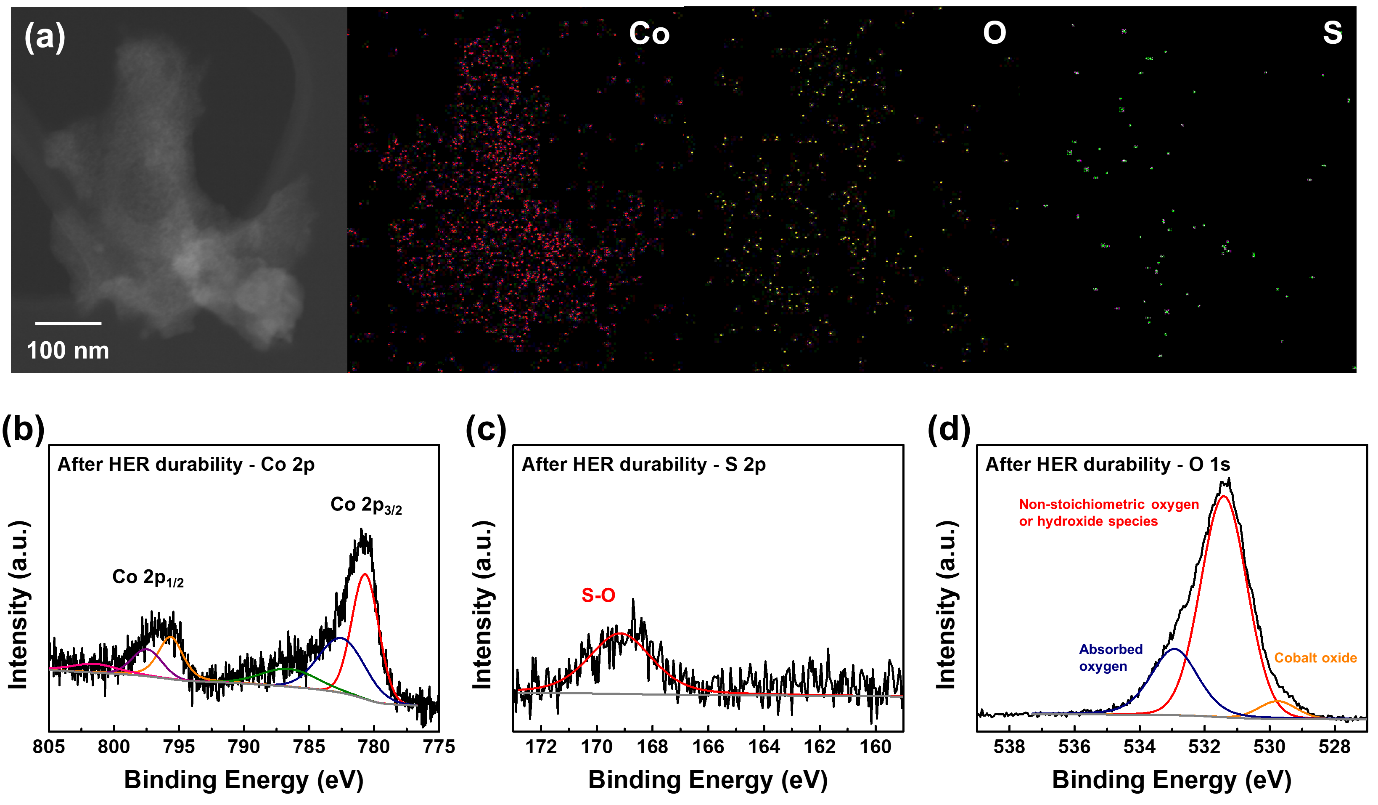


**Figure S15.** Chemical composition and state change of MOF-driven CoS_2_ after 20 h HER durability at -0.2 V (vs. RHE). (a) STEM image and EDX elemental mapping images (red: Co, yellow: O, green: S in inset). (b-d) XPS analysis. (b) Cobalt 2p, (c) sulfur 2p, and (d) oxygen 1s spectrum.

**Table S1.** Comparison of OER catalytic activity with previous reports of transition metal compounds.

| **Catalyst** | **Electrolyte** | **Overpotential**  **(mV)** | **Current density**  **(mA cm^-2^)** | **Tafel slopes**  **(mV dec^-1^)** | **Substrate** | **Ref.** |
| --- | --- | --- | --- | --- | --- | --- |
| MOF-driven CoS_2_ | 1.0 M KOH | 298 mV | 10 mA cm^-2^ | 94 mV dec^-1^ | Ni foam | Current work |
| CoS_2_ microboxes | 1.0 M KOH | 308 | 10 | 41.4 | Glassy carbon | S6 |
| Porous (Ni_0.33_Co_0.67_)S_2_ | 1.0 M KOH | 295 | 100 | 78 | Carbon cloth | S7 |
| Pt-CoS_2_ | 1.0 M KOH | 300 | 10 | 58 | Carbon cloth | S8 |
| Co@NC, (core-shell) | 1.0 M KOH | 330 | 10 | 43.9 | Ni foam | S9 |
| Co-Mo-Nitride | 1.0 M KOH | 294 | 10 | 57 | Glassy carbon | S10 |
| NiCoP/C nanoboxes | 1.0 M KOH | 330 | 10 | 96 | Glassy carbon | S11 |
| Co/CoP | 1.0 M KOH | 340 | 10 | 79.5 | Glassy carbon | S12 |
| Au@CoS_x_,  (core-shell) | 0.1 M KOH | 345 | 10 | 138 | Glassy carbon | S13 |
| CoZn-Se | 1.0 M KOH | 320 | 10 | 66 | Glassy carbon | S14 |
| Co, N doped porous carbon nanosheet | 1.0 M KOH | 350 | 10 | 83 | Glassy carbon | S15 |
| 2D nonlayered NiSe | 1.0 M NaOH | 290 | 10 | 77.1 | Ni foam | S20 |
| Co(S_0.71_Se_0.29_)_2_ in carbon fiber | 1.0 M KOH | 307 | 10 | 67.5 | Ni foam | S21 |
| CoS_2_ in carbon fiber | 1.0 M KOH | 386 | 10 | 81.4 | Ni foam | S21 |

**Table S2.** Comparison of HER catalytic activity with previous reports of transition metal compounds.

| **Catalyst** | **Electrolyte** | **Overpotential**  **(mV)** | **Current density**  **(mA cm^-2^)** | **Tafel slopes**  **(mV dec^-1^)** | **Substrate** | **Ref.** |
| --- | --- | --- | --- | --- | --- | --- |
| MOF-driven CoS_2_ | 1.0 M KOH | -196 mV | -10 mA cm^-2^ | 113 mV dec^-1^ | Ni foam | Current work |
| Porous (Ni_0.33_Co_0.67_)S_2_ | 1.0 M KOH | -334 | -100 | 127 | Carbon cloth | S7 |
| Co/CoP | 1.0 M KOH | -253 | -10 | 73.8 | Glassy carbon | S12 |
| CoS_2_-TiO_2_ | 1.0 M KOH | -198 | -10 | 55 | Glassy carbon | S16 |
| Carbon tubes / Cobalt sulfide | 1.0 M KOH | -190 | -10 | 131 | Carbon paper | S17 |
| NiS_2_ hollow microsphere | 1.0 M KOH | -219 | -10 | 157 | Glassy carbon | S18 |
| Ni_0.7_Fe_0.3_S_2_ microflowers | 1.0 M KOH | -155 | -10 | 109 | Ni foam | S19 |
| 2D nonlayered NiSe | 1.0 M NaOH | -177 | -10 | 58.2 | Ni foam | S20 |
| Co(S_0.71_Se_0.29_)_2_ in carbon fiber | 1.0 M KOH | -122 | -10 | 85.7 | Ni foam | S21 |
| CoS_2_ in carbon fiber | 1.0 M KOH | -233 | -10 | 150.2 | Ni foam | S21 |
| FeS_2_/C nanoparticles | 1.0 M KOH | -202 | -10 | 98 | Ni foam | S22 |

**Table S3.** Comparison of overall water splitting performances with previous reports of transition metal compounds.

| **Catalyst** | **Electrolyte** | **Voltage (V)** | **Current density**  **(mA cm^-2^)** | **Substrate** | **Ref.** |
| --- | --- | --- | --- | --- | --- |
| MOF-driven CoS_2_ | 1.0 M KOH | 1.65 V | 10 mA cm^-2^ | Ni foam | Current work |
| Porous (Ni_0.33_Co_0.67_)S_2_ | 1.0 M KOH | 1.57 | 10 | Carbon cloth | S7 |
| Carbon tubes / Cobalt sulfide | 1.0 M KOH | 1.743 | 10 | Carbon paper | S17 |
| Ni_0.7_Fe_0.3_S_2_ microflowers | 1.0 M KOH | 1.625 | 10 | Ni foam | S19 |
| 2D nonlayered NiSe | 1.0 M NaOH | 1.69 | 10 | Ni foam | S20 |
| Co(S_0.71_Se_0.29_)_2_ in carbon fiber | 1.0 M KOH | 1.63 | 10 | Ni foam | S21 |
| FeS_2_/C nanoparticles | 1.0 M KOH | 1.72 | 10 | Ni foam | S22 |
| TiO_2_@Co_9_S_8_ array | 1.0 M KOH | 1.56 | 10 | Carbon rod | S23 |
| Co_9_S_8_ array | 1.0 M KOH | 1.71 | 10 | Carbon rod | S23 |
| Porous NiFe/NiCo_2_O_4_ | 1.0 M KOH | 1.67 | 10 | Ni foam | S24 |

**References**

S1 Xu, X., Song, F. & Hu, X. A nickel iron diselenide-derived efficient oxygen-evolution catalyst. *Nat Commun* **7**, 12324, doi:10.1038/ncomms12324 (2016).

S2 Deng, H. *et al.* Laser induced MoS2/carbon hybrids for hydrogen evolution reaction catalysts. *Journal of Materials Chemistry A* **4**, 6824-6830, doi:10.1039/c5ta09322h (2016).

S3 Wang, D., Wang, J., Luo, X., Wu, Z. & Ye, L. In Situ Preparation of Mo2C Nanoparticles Embedded in Ketjenblack Carbon as Highly Efficient Electrocatalysts for Hydrogen Evolution. *ACS Sustainable Chemistry & Engineering* **6**, 983-990, doi:10.1021/acssuschemeng.7b03317 (2017).

S4 Shit, S. *et al.* Cobalt Sulfide/Nickel Sulfide Heterostructure Directly Grown on Nickel Foam: An Efficient and Durable Electrocatalyst for Overall Water Splitting Application. *ACS Appl Mater Interfaces* **10**, 27712-27722, doi:10.1021/acsami.8b04223 (2018).

S5 McCrory, C. C. *et al.* Benchmarking hydrogen evolving reaction and oxygen evolving reaction electrocatalysts for solar water splitting devices. *J Am Chem Soc* **137**, 4347-4357, doi:10.1021/ja510442p (2015).

S6 Hua, Y., Jiang, H., Jiang, H., Zhang, H. & Li, C. Hierarchical porous CoS 2 microboxes for efficient oxygen evolution reaction. *Electrochimica Acta* **278**, 219-225, doi:10.1016/j.electacta.2018.05.028 (2018).

S7 Zhang, Q. *et al.* Self-Interconnected Porous Networks of NiCo Disulfide as Efficient Bifunctional Electrocatalysts for Overall Water Splitting. *ACS Appl Mater Interfaces* **10**, 27723-27733, doi:10.1021/acsami.8b04386 (2018).

S8 Han, X. *et al.* Ultrafine Pt Nanoparticle-Decorated Pyrite-Type CoS2 Nanosheet Arrays Coated on Carbon Cloth as a Bifunctional Electrode for Overall Water Splitting. *Advanced Energy Materials* **8**, 1800935, doi:10.1002/aenm.201800935 (2018).

S9 Sivanantham, A. *et al.* A Stable Graphitic, Nanocarbon-Encapsulated, Cobalt-Rich Core-Shell Electrocatalyst as an Oxygen Electrode in a Water Electrolyzer. *Advanced Energy Materials* **8**, 1702838, doi:10.1002/aenm.201702838 (2018).

S10 Chu, H., Zhang, D., Jin, B. & Yang, M. Impact of morphology on the oxygen evolution reaction of 3D hollow Cobalt-Molybdenum Nitride. *Applied Catalysis B: Environmental* **255**, 117744, doi:10.1016/j.apcatb.2019.117744 (2019).

S11 He, P., Yu, X. Y. & Lou, X. W. Carbon-Incorporated Nickel-Cobalt Mixed Metal Phosphide Nanoboxes with Enhanced Electrocatalytic Activity for Oxygen Evolution. *Angew Chem Int Ed Engl* **56**, 3897-3900, doi:10.1002/anie.201612635 (2017).

S12 Xue, Z.-H. *et al.* Janus Co/CoP Nanoparticles as Efficient Mott-Schottky Electrocatalysts for Overall Water Splitting in Wide pH Range. *Advanced Energy Materials* **7**, 1602355, doi:10.1002/aenm.201602355 (2017).

S13 Mai, H. D., Le, V. C. T. & Yoo, H. Effective Fabrication and Electrochemical Oxygen Evolution Reaction Activity of Gold Multipod Nanoparticle Core–Cobalt Sulfide Shell Nanohybrids. *ACS Applied Nano Materials* **2**, 678-688, doi:10.1021/acsanm.8b01689 (2019).

S14 Fang, G. *et al.* Metal Organic Framework-Templated Synthesis of Bimetallic Selenides with Rich Phase Boundaries for Sodium-Ion Storage and Oxygen Evolution Reaction. *ACS Nano* **13**, 5635-5645, doi:10.1021/acsnano.9b00816 (2019).

S15 Tang, Y. *et al.* Cobalt and nitrogen codoped ultrathin porous carbon nanosheets as bifunctional electrocatalysts for oxygen reduction and evolution. *Carbon* **141**, 704-711, doi:10.1016/j.carbon.2018.09.080 (2019).

S16 Ganesan, P., Sivanantham, A. & Shanmugam, S. CoS2–TiO2 hybrid nanostructures: efficient and durable bifunctional electrocatalysts for alkaline electrolyte membrane water electrolyzers. *Journal of Materials Chemistry A* **6**, 1075-1085, doi:10.1039/c7ta09096j (2018).

S17 Wang, J., Zhong, H. X., Wang, Z. L., Meng, F. L. & Zhang, X. B. Integrated Three-Dimensional Carbon Paper/Carbon Tubes/Cobalt-Sulfide Sheets as an Efficient Electrode for Overall Water Splitting. *ACS Nano* **10**, 2342-2348, doi:10.1021/acsnano.5b07126 (2016).

S18 Tian, T., Huang, L., Ai, L. & Jiang, J. Surface anion-rich NiS2 hollow microspheres derived from metal–organic frameworks as a robust electrocatalyst for the hydrogen evolution reaction. *J. Mater. Chem. A* **5**, 20985-20992, doi:10.1039/c7ta06671f (2017).

S19 Yu, J., Cheng, G. & Luo, W. Ternary nickel–iron sulfide microflowers as a robust electrocatalyst for bifunctional water splitting. *Journal of Materials Chemistry A* **5**, 15838-15844, doi:10.1039/c7ta04438k (2017).

S20 Wu, H., Lu, X., Zheng, G. & Ho, G. W. Topotactic Engineering of Ultrathin 2D Nonlayered Nickel Selenides for Full Water Electrolysis. *Advanced Energy Materials* **8**, 1702704, doi:10.1002/aenm.201702704 (2018).

S21 Fang, L. *et al.* Tuning Unique Peapod-Like Co(SxSe1-x)2 Nanoparticles for Efficient Overall Water Splitting. *Advanced Functional Materials* **27**, 1701008, doi:10.1002/adfm.201701008 (2017).

S22 Li, Z. *et al.* Pyrite FeS2/C nanoparticles as an efficient bi-functional catalyst for overall water splitting. *Dalton Trans* **47**, 14917-14923, doi:10.1039/c8dt02927j (2018).

S23 Deng, S. *et al.* Hollow TiO2@Co9S8 Core-Branch Arrays as Bifunctional Electrocatalysts for Efficient Oxygen/Hydrogen Production. *Adv Sci (Weinh)* **5**, 1700772, doi:10.1002/advs.201700772 (2018).

S24 Xiao, C., Li, Y., Lu, X. & Zhao, C. Bifunctional Porous NiFe/NiCo2O4/Ni Foam Electrodes with Triple Hierarchy and Double Synergies for Efficient Whole Cell Water Splitting. *Advanced Functional Materials* **26**, 3515-3523, doi:10.1002/adfm.201505302 (2016).
